# Supplementary material for: The Anti-Cancer Effect of Mangifera indica L. Peel Extract is Associated to γH2AX-mediated Apoptosis in Colon Cancer Cells
Source: Antioxidants (Basel). 2019 Sep 22;8(10):422. doi: 10.3390/antiox8100422 (PMC6826946; doi:10.3390/antiox8100422)
Supplement: Supplementary file 1 [file antioxidants-08-00422-s001.pdf]

Supplementary Materials

The Anti-Cancer Effect of *Mangifera indica* L. Peel Extract is Associated to γH2AX-mediated Apoptosis in Colon Cancer Cells.

Marianna Lauricella, Valentina Lo Galbo, Cesare Cernigliaro, Antonella Maggio, Antonio Palumbo Piccionello, Giuseppe Calvaruso, Daniela Carlisi, Sonia Emanuele, Michela Giuliano and Antonella D'Anneo.

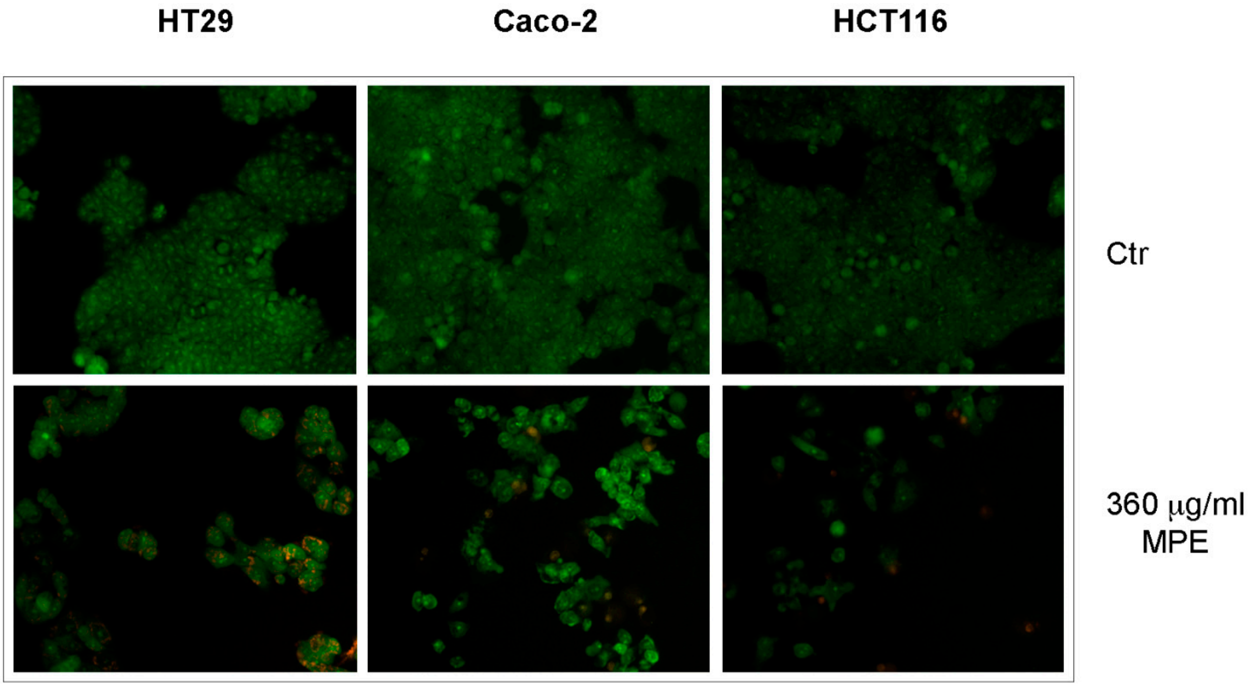

Figure S1. MPE treatment induces apoptotic cell death in colon cancer cells. Colon cancer cells were treated with MPE for 48 h, then cells were exposed to acridine orange/ethidium bromide dual staining as reported in Materials and Methods. Apoptotic cells were characterized by a granular yellow-green nuclear staining (early apoptotic cells) or condensed orange nuclear staining (late-apoptotic cells) compared to live cells (green fluorescent cells). Images were acquired using a Leica fluorescent microscope (original magnification 200X) and processed using Leica Q Fluoro software.

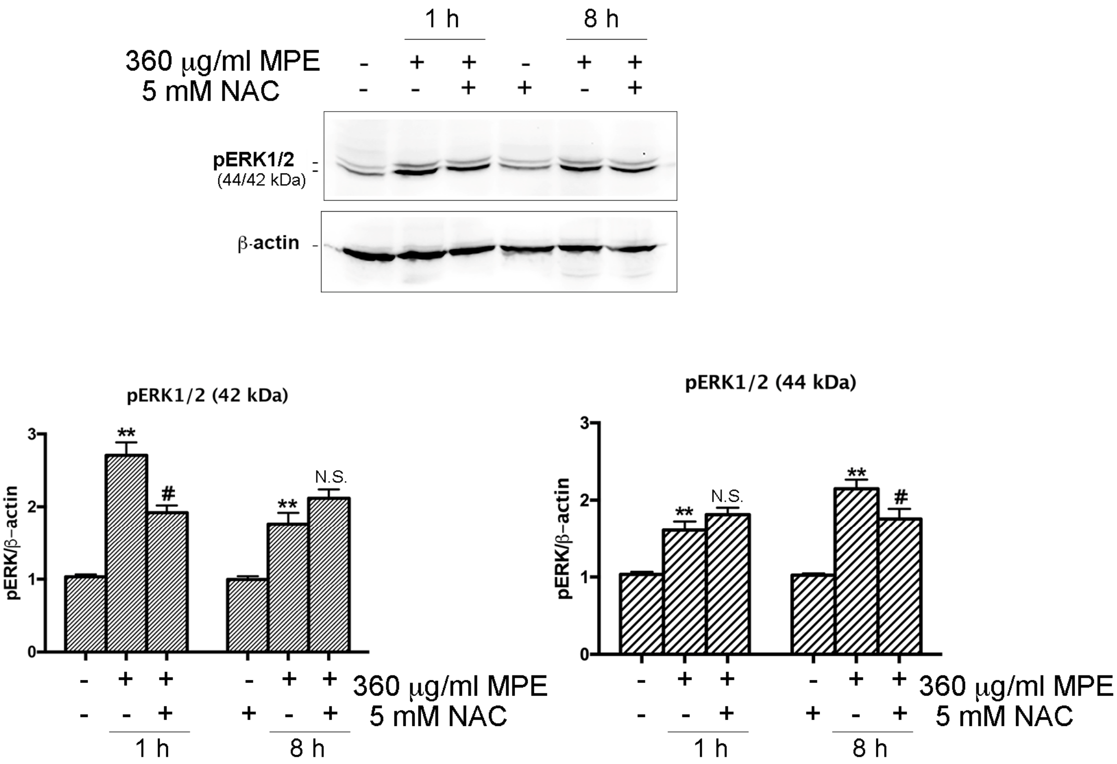

Figure S2. MPE treatment upregulates the main stress protein player pERK1/2. HT29 cells were treated with MPE for the indicated times in the presence or absence of 5 mM NAC. Lastly, western blotting analyses were performed in order to study MPE effects on the total level of pERK1/2 in the first phase of treatment (1-8 h). The correct protein loading was ascertained by immunoblotting for β-actin. The results are representative of three independent experiments and densitometry analysis histograms are reported normalized to β-actin. (**) $p < 0.01$ compared to the untreated sample. (#) $p < 0.05$ compared to MPE-treated sample. N.S., not significant in respect to MPE-treated cells.
